# The Internet as Scientific Knowledge Base: Navigating the Chem-Bio Space

**DOI:** 10.1002/minf.201200037

**Published:** 2012-08-07

**Authors:** Katrin Stierand, Tim Harder, Thomas Marek, Matthias Hilbig, Christian Lemmen, Matthias Rarey

**Affiliations:** a Center for Bioinformatics, University of Hamburg, Bundesstrasse 43, 20146 Hamburg, Germany; b BioSolveIT GmbH, An der Ziegelei 79, 53757 St. Augustin; c These authors contributed equally to this work

**Keywords:** Chemical space, Drug discovery, Medicinal chemistry, Molecular visualization, OpenPHACTS

The early phases of designing a new drug are characterized by an exhaustive exploration of related information available in public as well as in proprietary databases. Gathering such information for a given set of compounds is complicated due to the large number of data sources, the different data formats and query mechanisms used.^1^ Manual searches are tedious and time consuming, thus usually limited to individual compounds only. The process is error prone and the information collected this way is incomplete, of variable quality, and missing the link to the original data sources. Therefore data values can not necessarily be compared and it is difficult to identify compound duplicates.^2^ A recent approach to overcome these issues is the integration of data from different sources by means of semantic web technologies.^3^ Organizing the data in triples allows the representation of relationships between data points. Here, each triple has a subject-predicate-object structure, i.e. “sodium chloride (=subject) is a (=predicate) salt (=object)”. This way, related pieces of information can be connected semantically. Next to assembling the data itself, another challenging aspect is the representation of the data in a user-friendly and comprehensible manner. The constantly growing amount of data to be considered in a single drug discovery process poses new challenges to find a human accessible representation. The visualization of compound structures along with the related textual information should be designed for interactive use and at the same time provide access to important details. Commonly used cheminformatics tools for the handling of large sets of compounds include Spotfire (TIBCO),^4^ MS Excel,^5^ Accord (Accelrys),^6^ the Dotmatics Browser,^7^ Seurat (Schrödinger),^8^ CDD Vault,^9^ and Tableau.^10^ These tools allow exploring entire datasets, e.g. by plotting numerical properties against each other or by presenting them in a tabular form. However, the compound information cannot be augmented dynamically, but is instead restricted to the information contained in the original dataset.

Employing the Internet as a knowledge base entails different challenges. The available data is spread over numerous sources, which are updated frequently but not necessarily regularly. Due to this dynamic nature of data, downloading a copy and working with it locally quickly leads to outdated, false or simply missing information. Other, more technical challenges are the large amounts of data, the diverse and often non-standard formats along with the use of different molecule identifiers in most data sources. In the context of semantic web technology, this can be solved by so-called identity resolution services (IRS),^11^ which are entrusted with the mapping of different identifiers to one unique object representation.

In 2011 a new project, called OpenPHACTS (Open Pharmacological Concept Triple Store),^12^ has been initiated aiming to create an Open Pharmacological Space (OPS) by assembling data useful for drug discovery from public data sources using semantic web technologies. Currently, it includes datasets from the following databases: DrugBank,^13^ ChEMBL,^14^ SwissProt/UniProt,^15^ ChEBI,^16^ Gene Ontology,^17^ GOA,^18^ Wikipathways.^19^ Following an application-oriented approach, the project started with the definition of potential use cases in the form of research questions, formulated and prioritized by a consortium of scientists from both academia as well as various pharmaceutical companies.^20^ The development of the different software components is now led by these research questions.

So-called Exemplar Services are an important part of the OpenPHACTS project, show-casing the versatile application possibilities of OPS. These web services are using the OPS framework and provide access to the data in a user friendly manner. Each exemplar focusses on a certain application domain, covering target-related, polypharmacology as well as compound-related research questions.^20^ The exemplar services should enable the average bench scientist to access the data in an intuitive fashion with minimal learning effort and particularly avoiding the necessity to use complicated query languages such as JavaScript/JSON or SPARQL.^21^

Here we present a first prototype of the ChemBioNavigator (CBN), an OpenPHACTS exemplar service for navigating the chem-bio space with a focus on small molecules relevant in pharmaceutical research. This service allows to access large amounts of data originating from numerous public data sources available on the Internet and to merge this with proprietary compound information dynamically during runtime. The added information is taken directly from datasets included in the OPS or from external data sources which are referenced in the OPS data cache.

Taking a step beyond the use-case driven development, the CBN is based on the analysis of several interviews with potential users from pharmaceutical industry. The interviews gave invaluable insights not only about the day to day use-cases, but also deficiencies as well as highly valued features of the existing tools. While the requirements of users may be competing, thus can never all be satisfied, trends usually become apparent as the number of interviewed parties grows. This leads to an agile and target-oriented development, which is able to quickly adapt to changing requirements from the scientist’s daily work.

The CBN is realized using modern web technologies and state of the art cheminformatics software libraries. The user interface consists of two different regions: A large molecule visualization canvas and an information panel (see Figure 1).

**Figure 1:**
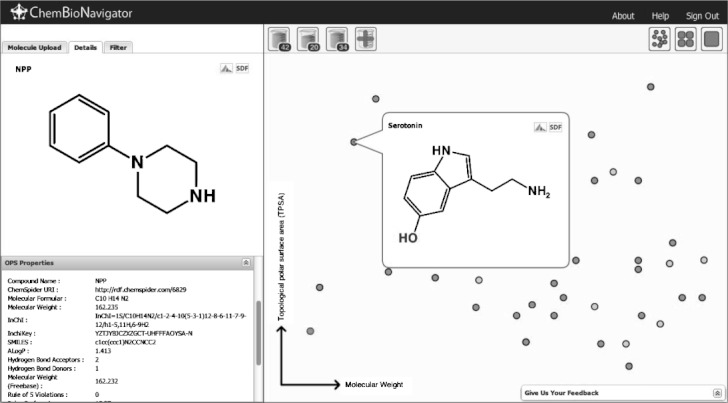
The ChemBioNavigator user interface consists of two areas: the information panel on the left hand side and the visualization area on the right hand side. When a compound is selected in the drawing area, it is highlighted by a green circle and the available details are displayed in the information panel. In this figure, properties of 1-Phenylpiperazine obtained from the OPS system are shown along with the structure diagram. The corresponding data point in the drawing area is located in its lower left corner. On mouse over, a preview of the compound structure diagram is displayed in a tooltip, as shown for serotonin in this example.

A CBN-session starts by uploading molecules either as a SD file or a mol2 file or in the form of SMILES strings. These are processed and validated using the NAOMI^22^ software library. NAOMI also calculates certain base-properties, such as the molecular weight, the number of hydrogen bond donors and acceptors, or a calculated logP. Subsequently the compounds are annotated with information obtained from the OPS system. These additional information range from identifiers and one-dimensional compound representations, such as InChI^23^ and SMILES^24^ strings, over topological and physico-chemical properties, to target-relations and assay data. Compound data uploaded from file is displayed and available for analysis as well.

For data analysis purpose, the entire compound set can be drawn in a scatter-plot on the visualization canvas. The user can choose any numerical property from any of the incorporated data sources to sort-order the data points via the *x*- and *y*-axis of the scatter plot. The data is color-coded based on whether the system was able to identify the compound within OPS and was able to obtain additional information. As known from browser based map services, it is possible to move through the scatter-plot using the mouse as well as to zoom in and out. When zooming in, the level of detail increases and mere dots are replaced by the actual molecule depictions. Additionally, a tabular representation of structure diagrams and the detailed view of single compounds are available allowing a closer examination of selected molecules. From the set of compounds, subsets can be selected and saved as SD file for further use. The SD file covers all compound data including the information retrieved from the OPS platform. The information panel is subdivided into the following tabs: The details-tab shows the structure diagram of the currently selected compound and lists all available properties. The properties are grouped as base properties assigned during the initialization process, properties provided in the uploaded file and additional properties originating from the OPS platform. The selection of properties for the axes of the scatterplot is visually supported by histograms, generated on the fly showing the value range and distribution of the currently chosen property.

Using the OPS system as data source, the CBN provides homogenous access to heterogeneous data originating from various databases. The functionality described here constitutes a first prototype of the CBN, which will be extended with various additional features over the course of the OpenPHACTS project. The main focus of further development will be the inclusion of target- and biological data. Given the project-focus on pharmaceutical research, the relationship between compound and potential targets is of particular interest. However, rather than duplicating functionality, links into other exemplars shall be provided to address very detailed or more complex research questions from other domains. Another important feature for future releases is the possibility to extend a compound set based on similarity searches.

The incorporation of these and other new features will enable the average bench scientist to easily explore large amounts of data, augmenting their research results with new aspects. The CBN is made publicly available at http://cbn.zbh.uni-hamburg.de.

## Computational Methods

**Figure 2:**
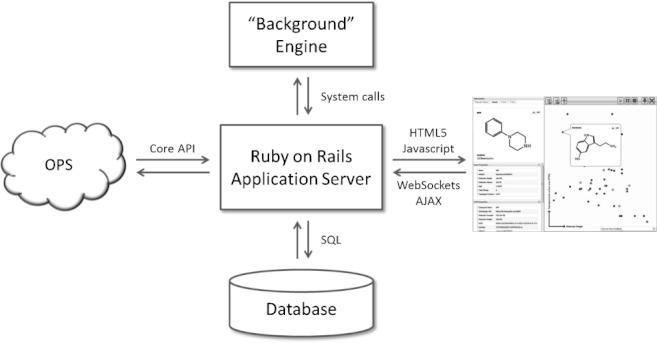
Schematic overview of the ChemBioNavigator technical architecture. The central application server connects the different parts of the system and handles the client communication.

Technically, the main challenges in this project were to design an architecture that is able to handle remote querying of the OPS system and at the same time provide an intuitive and responsive user interface. Further, the architecture should be flexible enough to be able to adapt to possible future request such as additional data sources like proprietary algorithms or databases. The CBN architecture is designed around a Ruby on Rails^25^ web-application server, connecting the different parts of the application. Next to the task of serving the webpages, the Rails server also handles the remote OPS queries, connects to the local database as well as manages the so-called background engine, responsible for the initialization and validation of compound (see Figure 2).

Currently the background engine mainly constitutes of the NAOMI^22^ cheminformatics library. Uploaded molecule files passed on from the Rails server are chemically validated and annotated with basic properties. This information is stored in a dedicated database directly on the server. In a separate step, two-dimensional structure diagrams are calculated for each molecule using an in-house layout algorithm based on the methods proposed by Helson.^26^ The validated structure diagrams along with the chemical properties are sent back to the client browser and are immediately available for browsing, sorting and exporting. The modular architecture then allows for retrieving information from the OPS core platform in an independent process, while browsing the available data. The challenge of the retrieval process is to uniquely identify the compound in question. This can either be achieved by specifically passing a compound identifier along with the structure information when uploading the file via a specific keyword. As a fallback mechanism, a structure matching against the large ChemSpider^27^ database is used. Only if both fail, no information can be retrieved. With the unique identifier, the OPS platform can be queried and additional information retrieved and stored along with the imported and the calculated base properties in the database. The result of this step is also send to the browser client using the popular Faye messaging system^28^ and is henceforth available for visualization.

During the development of the CBN, we focused on the application of modern web technologies such as HTML5, JavaScript and AJAX/JSON. The molecule visualization pane for example is realized solely using the HTML5 canvas element along with extensive JavaScript. The communication between the client and the server is largely based on WebSockets, facilitating bidirectional communication, i.e. the server is able to notify the client about newly obtained query results. The advantages of using standardized technologies over application frameworks such as Adobe Flash or Microsoft Silverlight are that, next to a web browser, no further software needs to be installed on the client computer. The latter was also mentioned as one of the obstacles the end-users reported during the interviews with regards to existing software applications in their field.

Applying modern web technologies further allows the seamless integration across different platforms and even device types. Especially the growing amount of tablet computers and other mobile devices are readily able to access this web service. The information is thus accessible wherever needed, at the office computer or the tablet in a meeting or at the lab bench.
